# Antipsychotic Medication and Risk of Incident Seizure in People with Autism Spectrum Disorder: Analyses with Cohort and Within Individual Study Designs

**DOI:** 10.1007/s10803-021-05208-0

**Published:** 2021-11-09

**Authors:** Basmah H. Alfageh, Frank M. C. Besag, Le Gao, Tian-Tian Ma, Kenneth K. C. Man, Ian C. K. Wong, Ruth Brauer

**Affiliations:** 1 School of Pharmacy, University College London, London, UK; 2 College of Pharmacy, King Saud University, Riyadh, Saudi Arabia; 3 East London Foundation NHS Trust, Bedfordshire, UK; 4 Institute of Psychiatry, Psychology and Neuroscience, King’s College London, London, UK; 5 Centre for Safe Medication Practice and Research, Department of Pharmacology and Pharmacy, Li Ka Shing Faculty of Medicine, The University of Hong Kong, Pokfulam, Hong Kong; 6 Department of Medical Informatics, Erasmus University Medical Centre, Rotterdam, The Netherlands

**Keywords:** Autism spectrum disorder (ASD), Antipsychotic medication, Psychotropic medication, Incident seizure

## Abstract

There are many case reports of seizures apparently associated with the prescription of antipsychotics. This study aimed to examine whether there is an association between the prescription of antipsychotics and incident seizures in individuals with autism spectrum disorder using retrospective data based on patients’ chart review. A cohort study was conducted to compare the rate of incident seizure between 3923 users of antipsychotics with 10,086 users of other psychotropics. This was followed by a self-controlled case series (SCCS) analysis of 149 patients to eliminate the effect of time-invariant confounders. The results showed no evidence of increased risk of seizure after exposure to antipsychotic agents (Hazard Ratio 1.28, 95% CI 0.74–2.19) compared to other psychotropics.

**Supplementary Information:**

The online version contains supplementary material available at 10.1007/s10803-021-05208-0.

## Introduction

Autism spectrum disorder (ASD) is a neurodevelopmental disorder present at birth or beginning in early childhood (McPartland & Volkmar, 2012). In 2016, the prevalence of ASD reached 1.6 per 100 children in the UK (Alfageh et al., 2020). There is no cure for ASD; however, psychotropic medications are used to manage the neuropsychiatric comorbidities that often accompany it (Hsia et al., 2014; Ji & Findling, 2015; Wong et al., 2014).

Antipsychotics have commonly been used in the management of disruptive behaviours in individuals with ASD (Ji & Findling, 2015; Posey et al., 2008). The efficacy of antipsychotics in the management of behavioural disorder associated with ASD has been reported in several randomised controlled trials (RCTs) (Ghanizadeh et al., 2014; Ichikawa et al., 2017; McDougle et al., 2000; Nagaraj et al., 2006; Pandina et al., 2007). Risperidone and aripiprazole are antipsychotic medication approved in the USA by the Food and Drug Administration (FDA) for the treatment of irritability associated with autistic disorder in children (Owen et al., 2009; Shea et al., 2004). In the UK, risperidone has been approved for the management of behavioural disturbance in children and adolescents associated with ASD and conduct disorder (European Medicines Agency, 2007). However, many other antipsychotic medications are prescribed. A recent study on the psychotropic medication prescribing for patients with ASD using the UK primary care database found that antipsychotics was prescribed to approximately 12.4% of the treated cohort; 50.7% of the issued prescriptions were for risperidone and 49.3% for other antipsychotics (Alfageh et al., 2020).

Several published papers have described the adverse events reported with the use of these agents. Metabolic adverse events, such as weight gain and hyperprolactinemia, have been reported frequently (Almandil et al., 2013). Extrapyramidal symptoms (EPS), such as tardive dyskinesia (TD), have also been reported, particularly with the typical antipsychotics (Caroff et al., 2002; Posey et al., 2008).

Seizures are serious central nervous system (CNS) adverse events. Both first-generation and second-generation antipsychotics can lower the seizure threshold, increasing the chances of seizure occurrence (Hedges et al., 2003a, 2003b; Lertxundi et al., 2013). However, as highlighted in a previous review (Hedges et al., 2003a, 2003b), most of the literature in this area consists of case reports. There is a lack of well-designed analytical studies of the risk of seizures with antipsychotic medication, particularly in populations with ASD.

The situation is complicated by the fact that ASD itself and intellectual disability, which is common in people with ASD, are risk factors for seizures (Canitano, 2007; Volkmar & Nelson, 1990). The aim of this study was to determine the risk of incident seizure in a population of patients with ASD.

## Methods

### Data Source

IQVIA Medical Research Data (IMRD-UK) (formerly known as THIN) was the data source. This is a primary care electronic medical records database from the early 1990s to the present day. It covers approximately 6% of the UK population and has more than10.5 million patients, 3.7 million of them are actively registered patients. Data from IMRD-UK are generalisable to the UK for demographic structures and major condition prevalence (Blak et al., 2011). This database is validated as a source of data for use in pharmacoepidemiological research (Lewis et al., 2007) and has been utilised previously for the study of medication in ASD (Alfageh et al., 2020; Murray et al., 2014).

### Ethical Approval

Ethical approval for this fully anonymised study was obtained from the Scientific Review Committee (SRC), which was established to review research using the IMRD-UK database (ref: 18THIN044).

### Study Design

#### Cohort

**Fig. 1:**
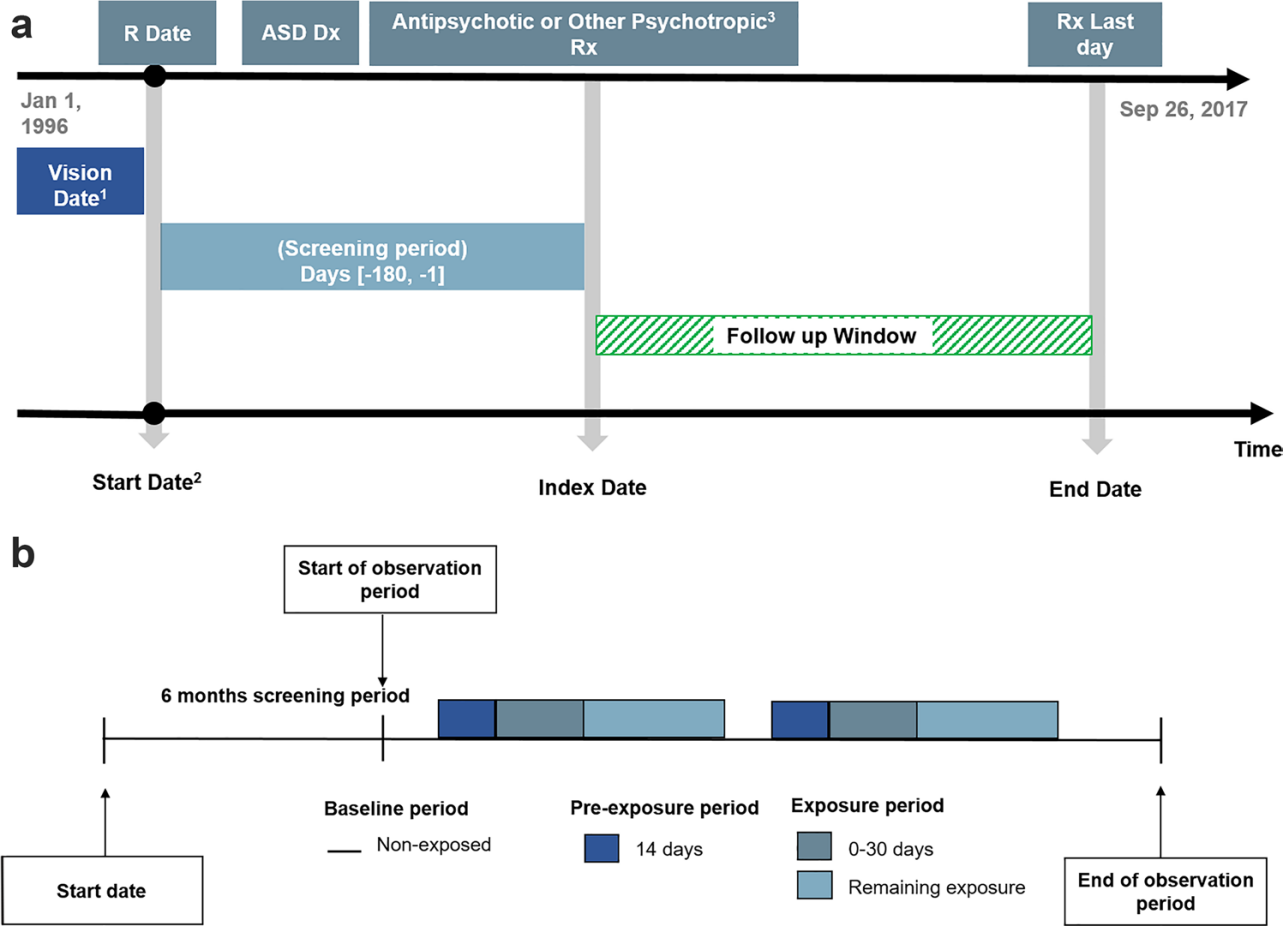
**a** Cohort analysis observation follow-up period. ^1^Vision Date is the date of implementing Vision software which is a computerised clinical management system used by the general practices to record patient information. ^2^Start Date is the latest of either the date of the individual patient registration at the general practice, Vision date, second birthday or the date of the study start Jan 1, 1996. ^3^Psychotropic medication classes included were: antidepressants, stimulants and non-benzodiazepine hypnotics and anxiolytics. ^4^The follow-up time of patients using ‘other psychotropic medication’ was censored once they received a prescription for an antipsychotic agent. An another follow-up period for them started on the first day of the antipsychotics prescription. *R Date* date of patient’s registration in the GP, *ASD Dx* autism spectrum disorder diagnosis, *Rx* drug prescription. **b** SCCS observation period

Two study designs were used in this retrospective study namely the cohort and the self-controlled case series (SCCS) design. In the cohort design, the risk of seizure in a population with ASD exposed to antipsychotics was compared to those who were on other psychotropic medication comprising antidepressants, stimulants or non-benzodiazepine hypnotics and anxiolytics. The exposure group was comprised of patients who had been prescribed antipsychotics after the diagnosis of ASD. Patients who had a record of epilepsy or seizure before the index date were excluded from the analysis. Some patients were exposed to both antipsychotics and other psychotropic medication. The follow-up time of patients using ‘other psychotropic medication’ was censored once they received a prescription for an antipsychotic agent. Another follow-up period for them started on the first day of the antipsychotics prescription (Fig. 1a).

In this cohort, patients were followed from the date of the first prescription of the study medication that followed the ASD diagnosis. This date was considered to be the index date for each patient. In the primary analysis, the end of the follow-up date was defined as the earliest of the following: occurrence of the outcome date, the medication of interest had been switched or discontinued, death, date of last data collection or the end date of the study. Figure 1a illustrates the follow-up period for each observation during the cohort primary analysis.

#### SCCS

An SCCS analysis was performed to estimate the incidence rate ratio (IRR) of seizure during predefined risk windows according to the exposure period compared to the remaining observation periods (non-exposure; baseline period) within each patient (see Fig. 1b). This study design overcomes the potential effect of time-fixed confounders such as genetic effects, as the comparison is carried out by using each individual as their own control (“self-controlled”) instead of comparing different groups of participants (Whitaker et al., 2006). In this SCCS analysis, the exposure was antipsychotic medication. Another SCCS analysis was conducted using other psychotropics specified in the cohort study (see appendix 1).

Seizure can be a recurrent event and the first seizure could be followed by other seizure events; therefore, only the first (incident) seizure was included in the analysis to avoid the violation of SCCS assumptions. A pre-exposure period was added to the risk period to consider the likelihood that the occurrence of seizure may affect the probability of antipsychotic treatment. During data extraction for the SCCS analysis, the data of the patients who had both an incident seizure and exposure to antipsychotics were extracted.

In this SCCS analysis, the observation start date was defined as the first day after the end of the six-month screening period. The ASD diagnosis date was recorded before or after the observation start, provided that it preceded or was on the date of first medication exposure. For each participant, the observation period was divided into the baseline period, including periods before and after medication exposure, and the exposure period. The exposure period was further divided into three risk windows: 14 days before medication exposure (the pre-exposure period); the first 30 days of medication exposure, and the subsequent medication exposure. Some participants had multiple intermittent medication exposures within their observation time: each continuous exposure was divided into three distinct risk windows. Figure 1b illustrates the observation period timeline for each participant during the SCCS analysis.

#### Participants, Exposure and Outcomes

Within the UK, the National Institute for Health and Care Excellence (NICE) guidelines follow the International Classification of Diseases, Tenth Revision (ICD-10) and the Diagnostic and Statistical Manual of Mental Disorders, Fifth Revision (DSM-5) criteria for ASD diagnoses(NICE, 2011). In the UK primary care databases, clinical information such as symptoms and diagnoses is recorded as coded data using Read codes. A medical dictionary for Read codes is available for researchers to develop a comprehensive set of condition-specific codes (code lists) which can be used to extract data that helps to identify cases or covariates of interest. Diagnostic Read codes were used to identify the patients in any age equal to or above two years with the first-recorded diagnosis of ASD between 1st of January 1996 to 26th September 2017 (see appendix 2). The start date of each patient was defined as the latest of the following: the date of the patient’s registration at the general practice, the date that the general practice began using Vision software (a clinical management system) or their second birthday. Patients were included if they received at least one prescription of the study medications, which included the following classes of psychotropic medication: antipsychotics, antidepressants, stimulants and non-benzodiazepine hypnotics and anxiolytics. Medication lists for each class were obtained from the British National Formulary Chapter 4 (see appendix 3). Anti-seizure medications (ASMs), (formerly known as antiepileptic drugs) and also benzodiazepines that are not necessarily listed as ASMs were not included because of the likely effect on the outcome of interest (seizures). Drug codes of the preceding psychotropics were extracted to identify medication exposure. Patients were considered eligible for inclusion in the study only if they had a screening period of at least six months available from their start date to the date of first prescription that followed the ASD diagnosis (except for those patients whose start date equalled their second birthday, for whom no screening period was required).

The outcome in this study was incident seizure. The seizure diagnosis was identified by the read codes list obtained from a previous study on incident seizure using a UK general practice database(Chui et al., 2016) (see appendix 4).

### Statistical Analyses (Cohort Study)

#### Propensity Score Fine-Stratification Weighting

Propensity score (PS) fine-stratification weighting with 50 strata was applied to adjust for potential confounders. Unlike conventional PS weighting such as Inverse probability of treatment weighting IPTW, PS fine-stratification does not depend directly on PS to calculate the observation weight; instead it uses PS to create fine strata. In each stratum, weights for the exposed group are set to 1 and un-exposed patients are reweighted based on the number of exposed patients residing within their stratum; Therefore, extreme weights resulting from PS that are close to 0 or 1 are unlikely (Desai & Franklin, 2019).

A number of potential confounders were included in the PS model (see appendix 5). Standardised mean differences (SMD) were used to examine the balance of covariates between the exposure groups (Table 1). SMD greater than 0.1 indicates evidence of imbalance between treated and control groups (Zhang et al., 2019). Hazard ratios (HR) of incident seizure were estimated using a Cox proportional hazard model. To adjust for potential clustering effect of patients contributed to both antipsychotics and other psychotropic groups, robust standard error was applied (Man et al., 2017).

**Table 1:** Characteristics baseline for the cohort study

Characteristic, no (%)	Crude		SMD	Weighted		SMD
	Antipsychotic	Other psychotropic^a^		Antipsychotic	Other psychotropic^a^	
Age, mean(SD)	25.7 (14)	18.1 (12.2)	0.582	25.7 (14)	26.7 (17.5)	− 0.061
Gender						
Female	906 (23.1)	2391 (23.7)	− 0.014	906 (23.1)	2353 (23.4)	− 0.006
Male	3017 (76.9)	7695 (76.3)	0.014	3011 (76.9)	7712 (76.6)	0.006
Smoking and alcohol status						
Current smoker	553 (14.1)	957 (9.5)	0.143	552 (14.1)	1508 (15)	− 0.025
Ex-smoker	265 (6.8)	659 (6.5)	0.009	265 (6.8)	832 (8.3)	− 0.057
Problematic drinker	203 (5.2)	362 (3.6)	0.078	203 (5.2)	605 (6)	− 0.036
Comorbidities						
Neuropsychiatric comorbidities ( +)	3346 (85.3)	7554 (74.9)	0.263	3340 (85.3)	8635 (85.8)	− 0.015
Diabetes ( +)	94 (2.4)	114 (1.1)	0.096	94 (2.4)	276 (2.7)	− 0.022
Hypertension ( +)	1079 (27.5)	2860 (28.4)	− 0.019	1079 (27.5)	3088 (30.7)	− 0.069
Stroke ( +)	8 (0.2)	10 (0.1)	0.027	8 (0.2)	21 (0.2)	0
Medication use						
Current user of Antidiabetic medication^b^	30 (0.8)	20 (0.2)	0.082	30 (0.8)	109 (1.1)	− 0.033
Ex-user of Antidiabetic medication^b^	9 (0.2)	9 (0.1)	0.035	9 (0.2)	15 (0.2)	0.018
Current user of Antihistamine	804 (20.5)	1761 (17.5)	0.077	802 (20.5)	2084 (20.7)	− 0.006
Ex-user of Antihistamine	698 (17.8)	2245 (22.3)	− 0.112	698 (17.8)	1861 (18.5)	− 0.017
Current user of Tramadol	27 (0.7)	88 (0.9)	− 0.021	27 (0.7)	82 (0.8)	− 0.014
Ex-user of Tramadol	39 (1)	115 (1.1)	− 0.014	39 (1)	137 (1.4)	− 0.034
Current user of NSAID	454 (11.6)	1192 (11.8)	− 0.008	454 (11.6)	1247 (12.4)	− 0.025
Ex- user of NSAID	617 (15.7)	2039 (20.2)	− 0.117	617 (15.8)	1629 (16.2)	− 0.012
Current user of Cytostatic	14 (0.4)	19 (0.2)	0.032	14 (0.4)	34 (0.3)	0.003
Ex-user of Cytostatic	6 (0.2)	18 (0.2)	− 0.006	6 (0.2)	15 (0.2)	0
Current user of Immunomodulator	7 (0.2)	22 (0.2)	− 0.009	7 (0.2)	18 (0.2)	0
Ex-user of Immunomodulators	6 (0.2)	11 (0.1)	0.012	6 (0.2)	38 (0.4)	− 0.043
ASM						
Current user of ASM	400 (10.2)	196 (1.9)	0.351	394 (10.1)	884 (8.8)	0.044
Ex-user of ASM	20 (0.5)	18 (0.2)	0.057	20 (0.5)	44 (0.4)	0.01
Current user of Benzodiazepine	451 (11.5)	272 (2.7)	0.348	446 (11.4)	1135 (11.3)	0.003
Ex-user of Benzodiazepine	49 (1.2)	64 (0.6)	0.064	49 (1.3)	154 (1.5)	− 0.024

*SMD* standardised mean difference, *SD* standard deviation, *NSAID* non-steroidal anti-inflammatory drug, *ASMs* anti-seizure medications

^a^Psychotropic medication classes included were: antidepressants, stimulants and non-benzodiazepine hypnotics and anxiolytics

^b^Antidiabetic medication included: glutathione and sulfonylurea

### Sensitivity Analyses (Cohort Study)

Sensitivity analyses were applied to examine the validity of the primary analysis. The purpose of these analyses was to investigate the effect of different follow-up periods on the resulting HR. In the sensitivity analyses, the definition of the end of follow-up date was changed to the following: (1) the earliest of: occurrence of the outcome date, death, the patient left the practice or the end date of the study; (2) the earliest of: occurrence of the outcome date, death, the patient left the practice or the end date of the study, 90 days after the first continuous medication exposure (grace period). The grace period was added to allow for the residual effect of the medication or the possibility of persistence administration form a residual supply of medication that had resulted from patient non-adherence.

### Statistical Analyses (SCCS)

A semi-parametric SCCS model was applied to estimate the risk by comparing the risk of incident seizure in different risk windows to the baseline period. In this model, the age effect does not need to be pre-specified (Farrington & Whitaker, 2006). Conditional Poisson regression was fitted to estimate the IRR, with 95%CIs.

### Sensitivity Analyses (SCCS)

In SCCS, if the occurrence of the outcome leads to the censoring of the observation, this will fail the assumption. A seizure episode could be serious and lead to death (although this would be a very unlikely event) which, subsequently, would end the observation. Although this would be an exceptionally rare event, we conducted a sensitivity analysis by excluding patients who had died during the study period.

An additional SCCS analysis was carried using a negative control outcome: a negative control outcome is a tool that is commonly applied in observational studies to examine the validity of the causal inferences (Lipsitch et al., 2010). It helps to detect selection and measurement bias in epidemiological studies (Arnold et al., 2016). The concept of this approach relies on looking for an association that cannot plausibly be hypothesised. The negative control outcome must share a common source of correlated measurement error with the true outcome (Arnold et al., 2016). Otitis media is an acute recurrent event; the occurrence of this event has never been linked with the use of antipsychotic medication. Therefore, it has been selected as the negative control outcome to validate the causal interpretations of the antipsychotics and incident seizure SCCS. Patients with ASD who had been exposed to antipsychotics and developed otitis media were included in the analysis. Patients with otitis media records before the observation period were excluded.

## Results

### Cohort

**Fig. 2:**
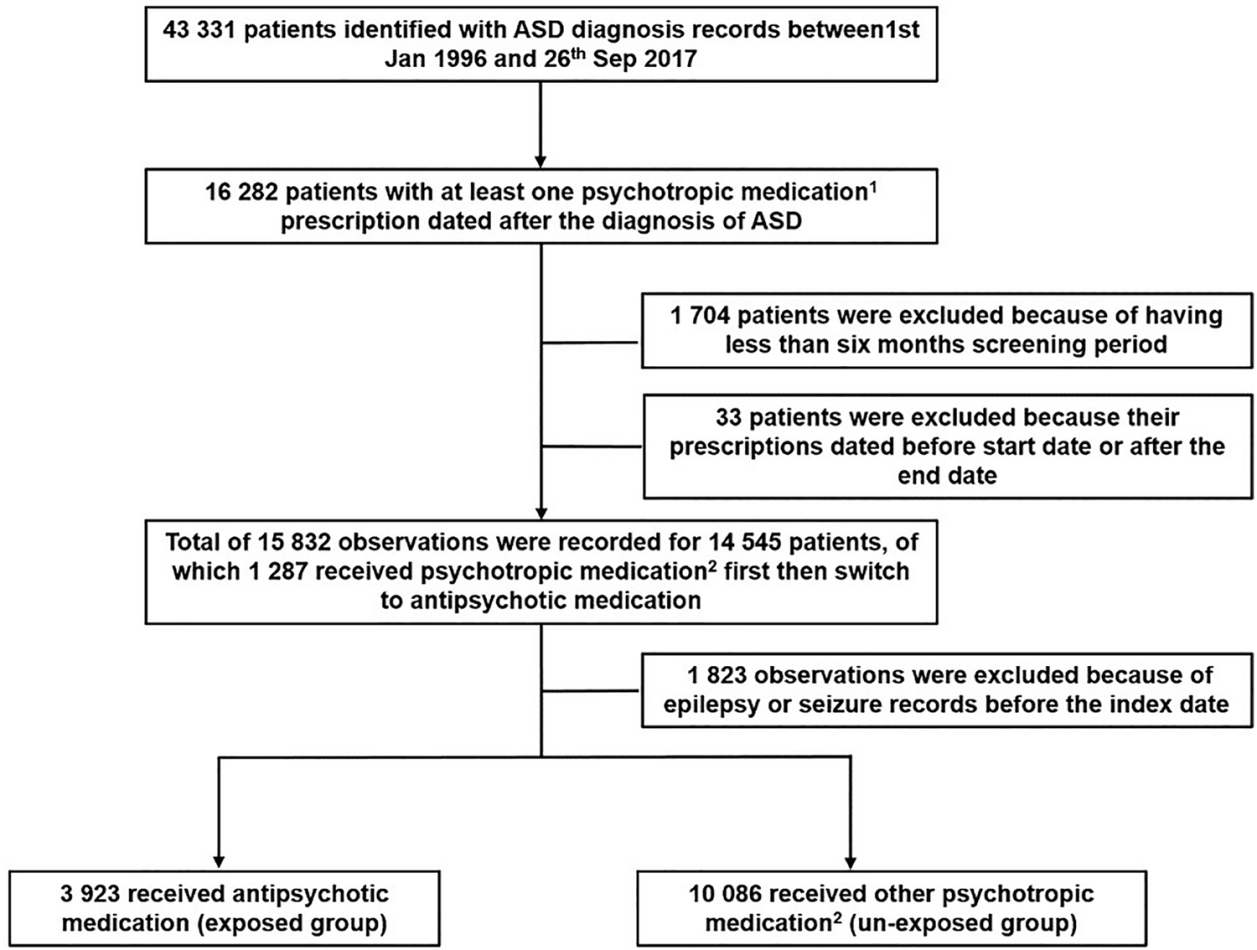
Flow chart for patients’ selection in the cohort study. ^1^In this step psychotropic medication classes included were: antipsychotics, antidepressants, anxiolytics, stimulants, and hypnotics, not including benzodiazepine. ^2^In this step psychotropic medication classes included were: antidepressants, stimulants and non-benzodiazepine hypnotics and anxiolytics

During the study period, we identified a total of 16,282 patients with ASD who had received at least one medication prescription of the study medications. Of these, 3560 patients were excluded because of having less than six months screening period, their prescriptions date before the start date or after the end date or having history of epilepsy or seizure. A total of 12,722 patients met the inclusion criteria and were included in the cohort analysis. 1287 patients received psychotropics first then switched to antipsychotics and were included in both groups to have a total of 14,009 observations of patients in the analysis. Figure 2 is a flowchart illustrating the patient selection process. Three thousand nine hundred and twenty-three patients receiving antipsychotic prescriptions were identified and allocated to the exposed group: 10,086 patients were identified as being on other psychotropic medication and they were considered to be the unexposed group. The mean age of the participants at the index date was 25.7 years (SD 14.0) for the exposed group and the mean follow-up was 2.2 years (SD 2.6). For the unexposed group, the mean age was 18.1 years (SD 12.2) and the mean follow-up was 3.0 years (SD 3.4). The ratio of male to female patients in both the exposed and unexposed groups was approximately 3:1. In the PS-weighted model, all covariates were balanced between the two study groups, with SMD less than 0.1. Table 1 lists the crude and weighted baseline clinical characteristics of the exposed and unexposed groups at the index date, with standardised mean difference.

In the primary analysis, the incidence of seizure was 54 per 10,000 person-years (PY) in 3923 patients using antipsychotic medication, 36 per 10,000 PY in 10 086 patients using other psychotropic medication. The PS-weighted HR of the incident seizure was 1.28, 95% CI 0.74–2.19, indicating no evidence of an increased risk of incident seizure associated with antipsychotic exposure compared to other psychotropics in the population with ASD. Sensitivity analyses results were consistent with the primary analysis; the HRs were 1.40, 95% CI 0.85–2.30 and 1.36 (0.72–2.57). Table 2 shows the results of the crude and weighted Cox proportional hazard model.

**Table 2:** Results of the cohort analyses

Group	Patients (n)	Patient-years	Incident seizures (n)	Crude HR (95% CI)	Weighted HR (95% CI)
Primary analysis					
Follow up end by earlier of: outcome date, medication has been switched or discontinued, death, patient left practice or study end date					
Antipsychotic	3923	11,914	65	1.59 (1.15–2.22)	1.28 (0.74–2.19)
Psychotropic^a^	10,086	22,577	82	1	1
Sensitivity analyses					
1. Follow up end by earlier of: outcome date, death, patient left practice or study end date					
Antipsychotic	3923	15,238	77	1.70 (1.26–2.30)	1.40 (0.85–2.30)
Psychotropic^a^	10,086	30,306	94	1	1
2. Follow up end by earlier of: outcome date, death, patient left practice, study end date or 90 days after first continuous exposure					
Antipsychotic	3923	8988	52	1.80 (1.23–2.65)	1.36 (0.72–2.57)
Psychotropic^a^	10,086	15,601	55	1	1

^a^Psychotropic medication classes included were: antidepressants, stimulants and non-benzodiazepine hypnotics and anxiolytics

### SCCS

One hundred and forty-nine patients were included in the SCCS analysis. The overall observation period was nearly 1529 patient-years. Almost 80% of the patients were males, with a mean age of 17.13 years (SD 14.59) at the start of observation. At the commencement of observation, the female patients were younger: the mean age of the females was 12.23 (SD 10.89). The average length of continuous antipsychotic prescriptions was 49 days, ranging from 1 to 2553 days. Table 3 provides details of the patient characteristics and the observation period.

**Table 3:** Patients characteristics in the SCCS analyses

Characteristic	No. of patients (%)	Age at observation start, mean (SD), Y	Length of prescription, median (range) [IQR], d	Risk period (exposure)		Baseline period (no exposure)	
				Incident seizures, No	Total follow-up time, patient-years	Incident seizures, No	Total follow-up time, patient-years
1. Risk of incident seizure associated with antipsychotic exposure							
All	149 (100)	16.15 (14.03)	49 (1–2553) [25–78]	61	479.4	88	1049.9
Male	119 (79.9)	17.13 (14.59)	50 (1–2553) [25–81]	53	408.7	66	795
Female	30 (20.1)	12.23 (10.89)	28 (1–471) [15–56]	8	70.7	22	254.9
2. Risk of incident seizure associated with antipsychotic exposure (excluding patients died during study period)							
All	147 (100)	15.90 (13.71)		60	469.2	87	1043.4
Male	117 (79.6)	16.84 (14.23)	50 (1–2553) [26–81]	52	398.5	65	788.5
Female	30 (20.4)	12.32 (11.51)	30 (1–1841) [27–65]	8	70.7	22	254.9
3. Risk of otitis media associated with antipsychotic exposure (negative control)							
All	334 (100)	13.44(13.77)	32 (1–3763) [16–71]	73	972.4	261	2691.6
Male	250 (74.8)	12.32 (13.26)	42 (1–3763) [21–74]	54	737	196	2042.3
Female	84 (25.1)	16.78 (14.75)	28 (2–3549) [14–60]	19	235.4	65	649.3

In the primary SCCS analysis, using a semi-parametric model, the IRR of seizure for the first 30 days of antipsychotic exposure was 1.79 (95% CI 0.97–3.30), which indicates no evidence of association between exposure to antipsychotics and increased risk of incident seizure. Two patients died during the study period and were excluded in the sensitivity analysis, the results of the sensitivity analysis were consistent with the primary analysis. During the three defined risk periods of the semi-parametric SCCS analysis for a negative outcome, the IRR indicated no evidence of an association between antipsychotic exposure and increased risk of otitis media. The results of the SCCS analyses are shown in Table 4.

**Table 4:** Results of semi-parametric self-controlled case series (SCCS) analyses

Risk window	Incident seizures (n)	Patient-years	Adjusted IRR (95% CI)
1. Primary analysis, antipsychotic medication exposure and risk of incident seizure			
Baseline period	88	1049.9	–
14 days pre antipsychotic exposure	9	57.5	1.66 (0.74–3.71)
First 30 days of antipsychotic exposure	26	156.3	1.79 (0.97–3.30)
Subsequent antipsychotic exposure	26	265.6	1.02 (0.53–1.96)
2. Sensitivity analysis, excluding patients died within observation period			
Baseline period	87	1043.4	–
14 days pre antipsychotic exposure	8	55.9	1.52 (0.65–3.58)
First 30 days of antipsychotic exposure	26	152.7	1.79 (0.96–3.35)
Subsequent antipsychotic exposure	26	260.6	1.08 (0.56–2.11)
3. Negative outcome control, antipsychotic medication exposure and risk of incident otitis media			
Baseline period	261	2691.6	–
14 days pre first antipsychotic exposure	8	119.5	0.74 (0.32–1.73)
First 30 days of antipsychotic exposure	23	306.1	0.77 (0.42–1.39)
Subsequent antipsychotic exposure	42	546.8	0.75 (0.42–1.34)

## Discussion

### Main Findings

This research found no evidence of an association between antipsychotic treatment and an increased risk of seizure in individuals with ASD. The PS-weighted cohort results found no evidence of increased risk of an incident seizure associated with antipsychotic exposure compared with the use of other psychotropic medication 1.28 (0.74–2.19). The results of the SCCS were consistent with the cohort study. The incidence rate ratio of seizure event was 1.79, 95% CI 0.97–3.30 during the first month of antipsychotic exposure.

Although there are published reports about antipsychotics and associated risk of seizures, most of these are descriptive studies. Therefore, a causal relation between antipsychotics use and development of seizure has not been unequivocally confirmed, particularly in individuals with ASD (Górska et al., 2019; Grover et al., 2015; Hedges et al., 2003a, 2003b; Williams & Park, 2015). It should be noted in the light of evidence that around 50% of the prescribed antipsychotics for individuals with ASD are not approved in the UK for use in this population, which may indicate an off-label medication prescribing (Alfageh et al., 2020).

The likelihood of the association between antipsychotics and seizures has been investigated in patients with schizophrenia, mood disorders and dementia. A nested case–control study conducted in the UK using the Clinical Practice Research Datalink (CPRD) found that the prescription of haloperidol, prochlorperazine or trifluoperazine was associated with an increased risk of seizures: adjusted odds ratio (OR) 2.51, 95% CI 1.51–4.18 compared with non-users (Bloechliger et al., 2015). However, the comparison was between antipsychotic users and non-users without specifying if the comparison group was on other-psychotropic medication or not and considering the study design used, the estimated risk could be inflated (Schuemie et al., 2019). In our study, we didn’t compare the risk of seizure between different antipsychotic medication. A study with data from the national health insurance research database (NHIRD) compared the risk of seizure among first and second-generation antipsychotics in patients diagnosed with schizophrenia and mood disorders (Wu et al., 2016). This study showed no evidence of a higher risk of seizure associated with first-generation antipsychotics than the second generation: HR 1.34, 95% CI 0.99–1.81; p = 0.06 (Wu et al., 2016). When compared to risperidone, clozapine HR 3.06, 95% CI 1.40–6.71; thioridazine HR 2.90, 95% CI 1.65–5.10; chlorprothixene HR 2.60, 95% CI 1.04–6.49 and haloperidol HR 2.34, 95% CI 1.48–3.71 all had a higher risk of antipsychotic-related seizure, while aripiprazole had a potentially lower risk of seizure: HR 0.41, 95% CI 0.17–1.00; p = 0.05 (Wu et al., 2016). However, the results of the previous study could be affected by confounding by indication rather than reflecting the actual effect of the medication on the risk of seizure. In our cohort study, most of the prescriptions of antipsychotics were for second-generation antipsychotics (82.4%) and 45% of the prescriptions were for risperidone. For patients included in the SCCS analysis, a higher percentage of the antipsychotics prescriptions were issued for risperidone (57.6%). In this study, we have not taken account of medication dose. There is considerable evidence from the literature that, for medications that are associated with increased seizure risk, the risk is very much related to medication dose (Górska et al., 2019; Grover et al., 2015; Varma et al., 2011). Other reports have suggested that low-dose antipsychotic medication, as used to treat anxiety and/or behavioural problems in young people with ASD might not be associated with an increased risk of seizures but this leaves the possibility that higher antipsychotic doses, such as those used to treat psychosis or bipolar disorder might be associated with an increased seizure risk.

### Strengths and Limitations

#### Strengths

To our knowledge, this is the first analytical study investigating the association between antipsychotic agents and incident seizure compared to other psychotropics in population with ASD. The source of the data used in this research is a large primary care database representative of the UK population. Two different study designs were applied; which allowed us to calculate the incidence rate of seizure associated with exposure to antipsychotics and to eliminate between-person variations.

The cohort study was used to estimate the HR of the incident seizure associated with antipsychotic exposure compared with other psychotropic medication. Both the number of ASD subjects identified in the number and eligible for our study (14,009 observations) were large; they were followed for an average of more than two years. The PS fine stratification model that was used adjusts the variability between the study groups. PS fine stratification is a newer approach of the standard PS weighting; this model provides smaller relative bias in estimates of cases of low exposure prevalence (Desai & Franklin, 2019).

The SCCS design that followed this analysis overcomes the effect of time-fixed measured and unmeasured potential confounders between individuals as each participant acts as their own control (Petersen et al., 2016). As the comparison of the event rate is within-person, a smaller sample size number is needed to conduct such a study. In our research, the case definition was very specific and was applied to a limited number of individuals. This sample involved individuals with ASD being treated with antipsychotics and had an incident seizure. The results of the sensitivity analysis were similar to the primary analysis. This indicated that the seizure events did not lead to death, which would subsequently have ended the observation; thus, no violation of the SCCS assumptions occurred during our study. This was consistent with the findings of another SCCS study (not on psychotropic medication) which applied the SCCS extension approach to examine the effects of seizure on censoring the observation period (Chui et al., 2016).

#### Limitations

IMRD-UK is a primary care database; therefore, only medication prescriptions provided by primary care general practitioners are recorded. Other prescriptions, for example, medication prescribed in secondary care settings or hospital discharge medication are not recorded. Similarly, the seizure diagnosis records; there could have been seizure events in hospital emergency departments that were not linked to the patients’ files in the general practice (GP). This could have led to an underestimate the number of cases. The results of this research apply only to individuals with ASD with no history of epilepsy or seizure, and to antipsychotics in general; the analysis as was not stratified by type of antipsychotic medication. As indicated in the discussion, we have not taken account of medication dose. There are indications from the literature that higher doses of psychotropic medications are more likely to precipitate seizures. In this study, dose stratified risk estimates could potentially result in biased findings affected by inadequate study power resulting from a limited sample size. This applies to the estimate of repeated seizures in the cohort design, while in the SCCS only incident seizure can be included to avoid the violation of the SCCS design assumptions. Therefore, these issues should be addressed further in future studies.

## Conclusion

People with ASD are at greater risk of developing seizures, whether treated with antipsychotic medication or not. No evidence of an increased risk of incident seizures associated with antipsychotics treatment in comparison with other psychotropics in the study population with ASD was identified. Future carefully-conducted studies in individuals with ASD and a history of epilepsy or seizures are recommended.

## Supplementary Information

Below is the link to the electronic supplementary material.

Supplementary file1 (DOCX 28 kb)

## Data Availability

No additional data are available for sharing.
