# Supplementary material for: Antipsychotic Medication and Risk of Incident Seizure in People with Autism Spectrum Disorder: Analyses with Cohort and Within Individual Study Designs
Source: J Autism Dev Disord. 2021 Nov 9;52(11):4817–27. doi: 10.1007/s10803-021-05208-0 (PMC9556371; doi:10.1007/s10803-021-05208-0)
Supplement: Supplementary file 1 — Supplementary file1 (DOCX 28 kb) [file 10803_2021_5208_MOESM1_ESM.docx]

## **Journal:**

Journal of Autism and Developmental Disorders.

## **Title:**

Antipsychotic medication and risk of incident seizure in people with autism spectrum disorder: Analyses with cohort and within individual study designs.

## **Authors:**

Basmah H Alfageh; Frank MC Besag; Le Gao; Tian-Tian Ma; Kenneth K. C. Man; Ian CK Wong; Ruth Brauer

## **Corresponding author:**

Dr. Ruth Brauer, PhD.

Research Department of Practice and Policy, School of Pharmacy University College London

BMA House Tavistock Square London WC1H 9JP, London, UK

E-mail: [r.brauer@ucl.ac.uk](mailto:r.brauer@ucl.ac.uk)

Tel: +44(0)20 7874 1273

**Appendix 1: Self-controlled case series analysis of psychotropic medication exposure and risk of incident seizure in individuals with Autism Spectrum Disorder (ASD).**

**Table 1** **Patients characteristics.**

| **Characteristic** | **No. of Patients (%)** | **Age at Observation start, Mean (SD), Y** | **Length of Prescription,**  **Median (Range) [IQR], d** | **Risk Window** | | **Baseline Window** | |
| --- | --- | --- | --- | --- | --- | --- | --- |
|  |  |  |  | **Incident seizures, No.** | **Total Follow-up Time, Patient-years** | **Events, No.** | **Total Follow-up Time, Patient-years** |
| 1. **Risk of incident seizure associated with other psychotropic exposure*.** | | | | | | | |
| **All** | 305 (100) | 11.52(12.29) | 30(1-2367) [15-60] | 97 | 843.5 | 208 | 2,325 |
| **Male** | 230 (75.4) | 11.27(12.55) | 30(1-2367) [15-60] | 71 | 675.1 | 159 | 1,752.9 |
| **Female** | 75 (24.6) | 12.32 (11.51) | 30(1-1841) [27-65] | 26 | 168.4 | 49 | 572.1 |

*Psychotropic medication classes included were: antidepressants stimulants, anxiolytics and hypnotics, not including benzodiazepine.

**Table 2 Results of semi-parametric SCCS analysis**

| **Risk Window** | **Incident seizures (n)** | **Patient-years** | **Adjusted IRR (95% CI)** |
| --- | --- | --- | --- |
| **Baseline period** | 208 | 2,325 | - |
| **14 days pre first psychotropic exposure** | 18 | 111.1 | 1.57 (0.91-2.71) |
| **First 30 days of psychotropic exposure** | 42 | 275.4 | 1.57 (1.03-2.38) |
| **Subsequent psychotropic exposure** | 37 | 456.94 | 0.83 (0.53-1.32) |

**Appendix 2: ASD diagnosis Read codes list**

| **Read code** | **Description** |
| --- | --- |
| Eu84z11 | Autistic spectrum disorder |
| E140.12 | Autism |
| Eu84500 | Asperger's syndrome |
| Eu84011 | autistic disorder |
| E140.00 | infantile autism |
| Eu84000 | childhood autism |
| Eu84.00 | pervasive developmental disorders |
| Eu84100 | atypical autism |
| E140.13 | childhood autism |
| E140000 | active infantile autism |
| Eu84111 | atypical childhood psychosis |
| Eu84z00 | pervasive developmental disorder, unspecified |
| E140.11 | kanner's syndrome |
| E140100 | residual infantile autism |
| E140z00 | infantile autism nos |
| E141.00 | disintegrative psychosis |
| E141.11 | heller's syndrome |
| E141000 | active disintegrative psychoses |
| E141100 | residual disintegrative psychoses |
| E141z00 | disintegrative psychosis nos |
| Eu84012 | infantile autism |
| Eu84013 | infantile psychosis |
| Eu84014 | kanner's syndrome |
| Eu84112 | mental retardation with autistic features |
| Eu84300 | other childhood disintegrative disorder |
| Eu84311 | dementia infantalis |
| Eu84312 | disintegrative psychosis |
| Eu84313 | heller's syndrome |
| Eu84314 | symbiotic psychosis |
| Eu84400 | overactive disorder assoc mental retard/stereotype movts |
| Eu84511 | autistic psychopathy |
| Eu84y00 | other pervasive developmental disorders |

**Appendix 3: British National Formulary (BNF) list of psychotropic medication classes**

| **Antidepressant** | **Antiepileptic** | **Antipsychotic** | **Antipsychotic depots** | **Non-benzodiazepine Anxiolytic** | **Non-benzodiazepine Hypnotic** | **Stimulant** |
| --- | --- | --- | --- | --- | --- | --- |
| Agomelatine | Acetazolamide | Amisulpride | Aripiprazole | Bromazepam | Chloral hydrate | Amphetamine |
| Amitriptyline | Beclamide | Aripiprazole | Flupentixol | Buspirone | Clomethiazole | Ascorbic ac./cyanocob/ fencamfamin hyd/ |
| Amoxapine | Brivaracetam | Asenapine | Fluphenazine | Chlordiazepozide | Cloral betaine | Atomoxetine |
| Bolvidon | Carbamazepine | Benperidol | Fluspirilene | Chlormezanone | Dichloralphenazone | Caffeine |
| Butriptyline | Clobazam | Chlorpromazine | Haloperidol | Generic Kalms | Mandrax tab | Dexamfetamine |
| Citalopram | Clonazepam | Chlorprothixene | Olanzapine | Ketazolam | Melatonin | Dexamphetamine |
| Clomipramine | Diazepam | Clozapine | Paliperidone | Medazepam | Methyprylone | Dexbrompheniramine/pseudoephedrine |
| Desipramine | Epanutin | Dartalan | Pipotiazine | Meprobamate | Nitrados | Dexedrine |
| Dosulepin | Eslicarbazepine | Droperidol | Zuclopenthixol | Serenid | Promethazine | Guanfacine |
| Dothiepin | Ethosuximide | Flupentixol |  | Valerian | Sodium oxybate | Lisdexamfetamine |
| Doxepin | Fosphenytoin | Fluphenazine |  |  | Triclofos | Methylperidate |
| Duloxetine | Gabapentin | Haldol |  |  | Zaleplon | Methylphenidate |
| Escitalopram | Gardenal | Haloperidol |  |  | Zolpidem | Modafinil |
| Fluoxetine | Lacosamide | Levomepromazine |  |  | Zopiclone | Nicotin./prolintane hyd/pyridox.hyd/ribo |
| Fluvoxamine | Lamotrigine | Loxapine |  |  |  | Pemoline |
| Imipramine | Levetiracetam | Lurasidone |  |  |  | Prolintane |
| Iprindole | Mesuximide | Olanzapine |  |  |  | Reactivan |
| Iproniazid | Methsuximide | Oxypertine |  |  |  | Ronyl |
| Isocarboxazid | Methylphenobarbital | Paliperidone |  |  |  | Sodium oxybate |
| Lofepramine | Midazolam | Pericyazine |  |  |  | Tafamidis |
| L-tryptophan | Ospolot | Perphenazine |  |  |  |  |
| Maprotiline | Oxcarbazepine | Pimozide |  |  |  |  |
| Merital | Paraldehyde | Promazine |  |  |  |  |
| Mianserin | Paramethadione | Quetiapine |  |  |  |  |
| Mirtazapine | Pentamidine | Remoxipride |  |  |  |  |
| Moclobemide | Perampanel | Risperidone |  |  |  |  |
| Nefazodone | Phenobarbital | Sertindole |  |  |  |  |
| Nomifensine | Phenobarbitone | Sulpiride |  |  |  |  |
| Nortriptyline | Phenytoin | Thioproperazine |  |  |  |  |
| Paroxetine | Pregabalin | Thioridazine |  |  |  |  |
| Phenelzine | Primidone | Trifluoperazine |  |  |  |  |
| Protriptyline | Retigabine | Trifluperidol |  |  |  |  |
| Reboxetine | Rufinamide | Zotepine |  |  |  |  |
| Sertraline | Sodium valproate | Zuclopenthixol |  |  |  |  |
| Sinequan | Stiripentol |  |  |  |  |  |
| Tofranil | Sulthiame |  |  |  |  |  |
| Tranylcypromine | Tiagabine |  |  |  |  |  |
| Trazodone | Topiramate |  |  |  |  |  |
| **Antidepressant cont.** | **Antiepileptic**  **cont.** |  |  |  |  |  |
| Tranylcypromine | Valproic acid |  |  |  |  |  |
| Trimipramine | Vigabatrin |  |  |  |  |  |
| Tryptophan | Zarontin |  |  |  |  |  |
| Venlafaxine | Zonisamide |  |  |  |  |  |
| Viloxazine |  |  |  |  |  |  |
| Zimelidine |  |  |  |  |  |  |

| **Benzodiazepine Anxiolytic** | **Benzodiazepine Anxiolytic** |
| --- | --- |
| Alprazolam | Clorazepate |
| Chlordiazepoxide | Flunitrazepam |
| Clorazepate | Flurazepam |
| Diazepam | Lormetazepam |
| Oxazepam | Nitrazepam |
| Prazepam | Temazepam |
| Lorazepam | Triazolam |
| Clobazam |  |

**Appendix 4: Seizure diagnosis Read codes list**

| **Read code** | **Description** |
| --- | --- |
| 1B63.00 | Had a fit |
| 1B63.11 | Fit - had one, symptom |
| 1B64.00 | Had a convulsion |
| 1B64.11 | Convulsion - symptom |
| 282..00 | O/E - fit/convulsion |
| 282..11 | O/E - a convulsion |
| 282..12 | O/E - a fit |
| 282..13 | O/E - a seizure |
| 2822 | O/E - grand mal fit |
| 2823 | O/E - petit mal fit |
| 2824 | O/E - focal (Jacksonian) fit |
| 2824.11 | O/E - Jacksonian fit |
| 2824.12 | O/E - focal fit |
| 2825 | O/E - psychomotor fit |
| 2828 | Absence seizure |
| 282Z.00 | O/E - fit/convulsion NOS |
| Eu44500 | [X]Dissociative convulsions |
| F132y00 | Other specified myoclonus |
| F132z00 | Myoclonus NOS |
| F132z12 | Myoclonic seizure |
| F251600 | Grand mal seizure |
| F252.00 | Petit mal status |
| F253.00 | Grand mal status |
| F253.11 | Status epilepticus |
| F25C.00 | Drug-induced epilepsy |
| F25y300 | Complex partial status epilepticus |
| Fyu5200 | [X]Other status epilepticus |
| Fyu5900 | [X]Status epilepticus, unspecified |
| R003.00 | [D]Convulsions |
| R003100 | [D]Convulsions, infantile |
| R003200 | [D]Fit |
| R003211 | [D]Fit (in non epileptic) NOS |
| R003400 | [D]Nocturnal seizure |
| R003y00 | [D]Other specified convulsion |
| R003z00 | [D]Convulsion NOS |
| R003z11 | [D]Seizure NOS |
| Ryu7100 | [X]Other and unspecified convulsions |

**Appendix 5: Covariates included in PS model of the cohort study**

Several potential confounders were incorporated in the PS fine stratification model such as age, gender, smoking and problematic alcohol drinking. In addition, certain medical conditions were added to the adjustment model including neuropsychiatric comorbidities, diabetes, hypertension and stroke. Data on concomitant medication use was obtained for each patient for the following drugs/drug classes: non-steroidal anti-inflammatory drugs (NSAID), some antidiabetic medication, antihistamine, tramadol, cytostatic drugs, immunomodulators, ASMs. In both groups of the cohort study, patients may have concomitant prescriptions for ASMs; therefore, we had adjusted for the use of these medications in the weighted analysis.
